# Trans-Generational Effect of Maternal Lactation during Pregnancy: A Holstein Cow Model

**DOI:** 10.1371/journal.pone.0051816

**Published:** 2012-12-20

**Authors:** Oscar González-Recio, Eva Ugarte, Alex Bach

**Affiliations:** 1 Departamento Mejora Genética Animal, INIA, Madrid, Spain; 2 NEIKER-TECNALIA, Araba, Spain; 3 ICREA, Barcelona, Spain; 4 Department of Ruminant Production, IRTA, Caldes de Montbui, Spain; University of Connecticut, United States of America

## Abstract

Epigenetic regulation in mammals begins in the first stages of embryogenesis. This prenatal programming determines, in part, phenotype expression in adult life. Some species, particularly dairy cattle, are conceived during the maternal lactation, which is a period of large energy and nutrient needs. Under these circumstances, embryo and fetal development compete for nutrients with the mammary gland, which may affect prenatal programming and predetermine phenotype at adulthood. Data from a specialized dairy breed were used to determine the transgenerational effect when embryo development coincides with maternal lactation. Longitudinal phenotypic data for milk yield (kg), ratio of fat-protein content in milk during first lactation, and lifespan (d) from 40,065 cows were adjusted for environmental and genetic effects using a Bayesian framework. Then, the effect of different maternal circumstances was determined on the residuals. The maternal-related circumstances were 1) presence of lactation, 2) maternal milk yield level, and 3) occurrence of mastitis during embryogenesis. Females born to mothers that were lactating while pregnant produced 52 kg (MonteCarlo standard error; MCs.e. = 0.009) less milk, lived 16 d (MCs.e. = 0.002) shorter and were metabolically less efficient (+0.42% milk fat/protein ratio; MCs.e.<0.001) than females whose fetal life developed in the absence of maternal lactation. The greater the maternal milk yield during embryogenesis, the larger the negative effects of prenatal programming, precluding the offspring born to the most productive cows to fully express their potential additive genetic merit during their adult life. Our data provide substantial evidence of transgenerational effect when pregnancy and lactation coincide. Although this effect is relatively low, it should not be ignored when formulating rations for lactating and pregnant cows. Furthermore, breeding, replacement, and management strategies should also take into account whether the individuals were conceived during maternal lactation because, otherwise, their performance may deviate from what it could be expected.

## Introduction

The first stages of mammalian fetal development are determinant for the adult offspring, as dramatic changes in DNA methylation occur which are responsible of cell differentiation of the embryo [1]. This methylation process begins with primordial germ cells having very low methylation levels, then with gametogenesis parental imprinting tags are established, with substantially methylated but differing methylomes in the sperm and egg. In the pre-implantation early embryo, there is a wave of genome-wide demethylation that occurs rapidly in the paternal genome, except for centromeric, and comparative slowly in the maternal genome [2]. This is then followed by heavy *de novo* methylation, particularly in the somatic lineages as these are established. Lack of nutrients during pregnancy has been associated with an increased risk of suffering metabolic diseases in adult life. Some examples can be found in humans: mothers who were pregnant during famine in The Netherlands in 1944, what it is known as the ‘Hunger Winter’, had children and grandchildren with a wide range of health problems [3]. A similar pattern has been observed in sheep [4]. The maternal effects alter phenotypes in the offspring by changes in the epigenome during pregnancy and suckling [5], [6].

The adult dairy cow is unique in the sense that the first stages of fetal development coincide with maternal lactation. Lactation is a period with high metabolic demands which lead to mobilization of body energy reserves, and this metabolic environment is surrounding, in the dairy cow, a critical period for the epigenetic control of fetal development [7]–[10]. In the lactating and pregnant dairy cow, nutrients must be partitioned between the placenta and the mammary gland. Thus, it would seem reasonable to expect some epigenetic effects of high milk production on fetal development, and these could be partly acting as predisposing factors for the progressive increase of common metabolic diseases and complex traits observed in cattle [11], [12].

The Holstein cow is a breed that has been specifically selected for milk yield, producing more than 10,000 kg of milk per year. Conception typically occurs around lactation peak (within the first 3 months after the onset of lactation and former calving), and thus most (∼75%) of the gestation coincides with lactation. The lactation peak is a period characterized by some degree of negative energy balance, as cows are typically unable to consume sufficient nutrients to meet metabolic demands associated with high volumes of milk production. Current models used to estimate nutritional needs of dairy cattle do not take into account the nutrient needs of the conceptus until it reaches 190 d of fetal life [10]. We hypothesize that individuals whom fetal development occurred during maternal lactation have different prenatal programming than those individuals conceived in the absence of lactation, which may affect their future performance in adult life. For instances, there are some nutrients that have been shown to alter the methylation patterns in the embryo such as folic acid and methyl group donors [13], but at present time the potential needs of these nutrients to meet pregnancy requirements in dairy cattle are unknown (and ignored). Furthermore, since there is a large variability in milk yield in Holstein cattle, ranging from around 5,000 to 14,000 kg of milk per lactation, one would expect that the prenatal programming of individuals conceived in high productive cows, and thus within a more challenging metabolic scenario, would be greater than in those born to low producing animals [14], [15].

The objective of the current study was to determine the impact on adult life performance (productivity, lifespan, and metabolic efficiency) of the co-existence of fetal development with the maternal lactation in a specialized dairy breed as an animal model. It must be pointed out that Holstein breeding programs worldwide are subjected to exhaustive data recording, with large amount of pedigree, phenotypic and environmental information stored in a longitudinal manner, setting a great opportunity to study the environmental effects during prenatal programming in adult life.

## Results and Discussion

The effect of different maternal circumstances (**m**) during conception in the future adult individual was estimated using a Bayesian framework. Descriptive summary of number of individuals per number of maternal lactation, milk production and age at first calving of the cow, as well as the age at calving of her dam and her milk (305d standardized) production during pregnancy is given in Table 1. Phenotypic traits of milk yield (MY), fat/protein ratio in milk (FP) and functional lifespan (DIM) were previously adjusted by environmental and genetic effects: the additive genetic merit of the *i*-individual due to the action of the genes inherited from her parents via germ cells, and the environmental effects of age of the individual at first parity, combined effect of year-region at birth, and combined effect of herd-year of first parity (i.e. all other registered environmental effects different from those occurring within the maternal womb during the fetal period). The analysis of DIM included, additionally, the amount of milk (kg) produced during the first lactation as a covariate to adjust for culling due to low production of the cow. This sort of adjustment for lifespan has been referred to as functional longevity in the scientific literature [16], and it aims to reflect the ability of a cow not to be culled due to impaired fertility or severe metabolic problems. The environmental circumstances that were included in the model were chosen according to the models that are implemented worldwide in official genetic evaluations in many countries, adjusting simultaneously for genetic and environmental effects. These models serve to provide the genetic merit of individuals for breeding purposes [17].

**Table 1:** Number of cows per maternal lactation group, average standardized production and age at first calving of the cow generating the observation.

Group of maternal lactation	Number of observations	Age at calving (mo.)	Maternal age at calving (mo.)	Phenotypic milk production (kg/305 d)	Phenotypic maternal milk production (kg/305 d)
**0 (non-lactating mother)** 1	16,288	26.6 (3)	26.9 (3)	8680 (1583)	-
**1**	13,979	26.8 (3)	40.5 (4)	8543 (1566)	8174 (2258)
**2**	9,210	26.9 (3)	54.0 (5)	8448 (1522)	8630 (3489)
**3+**	5,588	26.9 (3)	67.0 (5)	8328 (1531)	8534 (3871)

Average maternal milk production within maternal group and age of the dam when the offspring was born. Standard deviations are given in parentheses.

1 individuals born from pregnancies in non lactating mothers (heifers) were grouped together (0), and all others were classified according to the tenth-percentiles of the milk production (kg) of the dam at conception.

### Effect of maternal lactation during lactation on the offspring

**Figure 1:**
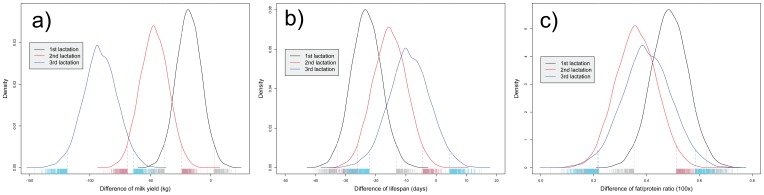
Effect of maternal lactation during embryogenesis on adult performance. The density plots show the posterior distribution of the difference in a) milk yield b) lifespan and c) fat/protein between cows whose embryogenesis occurred during the first (black line), second (red line) or third (blue line) maternal lactation and cows whom embryogenesis occurred in the absence of maternal lactation. The contrasts for presence vs absence of lactation showed lower production level, shorter lifespan and higher fat/protein ratio in the adult life of those individuals conceived during maternal lactation, with statistically significant posterior distributions.

The concurrence of pregnancy and maternal lactation has an effect (potentially through epigenetic regulation) on the fetus reducing future milk productivity. The posterior mean of the contrasts against non-concurrence of maternal lactation and their respective MonteCarlo standard error (MCs.e.) within parentheses were −18 (0.01), −47 (0.02) and −91 (0.02) kg of milk for the concurrence with 1^st^, 2^nd^ and 3^rd^ lactation, respectively (Figure 1.a). The highest probability density intervals (HPD95) of the posterior distributions were statistically different from zero and ranged between −38 and −1 for concurrence of 1^st^ lactation, between −69 and −24 for concurrence with second lactation, and between −119 and −64 for concurrence with 3^rd^ or subsequent lactations. Females that were conceived in the absence of maternal lactation produce more milk during the first lactation than daughters conceived during the maternal lactation with a probability greater than 0.98, regardless of the lactation number (1^st^, 2^nd^, 3^rd^ or subsequent) they were gestated in. Multiple reasons can be speculated for the observed negative effects on milk performance as the lactation number of the pregnant dam increases. For instance, it could be due to the increasing amount of milk produced in later lactations, accumulated unfavorable mutations in the germ line of the older dams, and/or a larger amount of epigenetic marks (that are transmitted to the embryo) being accumulated in older cows. This study does not provide evidence of potential genetic or epigenetic modifications in the fetus linked to increasing age of the cow, and future studies should tackle this hypothesis.

Females that were conceived in non-lactating dams had also longer lifespan than those whose mother was lactating during the first 220 d of pregnancy (Figure 1.b). In this case, the posterior mean estimates of the contrasts were 23 (MCs.e. = 0.003), 15 (MCs.e. = 0.003) and 9 (MCs.e. = 0.005) longer lifespan (in days) for individuals gestated in non-lactating mothers compared with those gestated during the first, second and third or subsequent lactations, respectively. The respective HPD95 were (13, 33), (4, 26) and (4,22). Females that were conceived in non-lactating mothers lived longer than those conceived during their mother's first, second and third or subsequent lactations with posterior probability of 1, 0.995 and 0.90, respectively.

An increased milk FP ratio is associated with poor metabolic efficiency and metabolic problems such as ketosis [18]–[20]. Milk FP ratio was larger in females whose fetal development occurred during their mother's lactation than in those born to heifers (non-lactating animals). The posterior distributions of the contrasts were larger than zero with probability greater than 0.999 and with posterior mean estimates equal to +0.49 (MCs.e.<0.001), +0.36 (MCs.e.<0.001) and +0.40 (MCs.e.<0.001) % for 1^st^, 2^nd^ and 3^rd^ and subsequent lactations, respectively (Figure 1.c).

Overall, these results indicate that the coincidence of pregnancy with lactation exerts some effect on the fetus, with a probable degree of cell programming, causing long-term effects on milking performance and metabolic efficiency, and thus longevity. Heterogeneity for growth rate in the heifers groups was not accounted for, and there could be some additional impact of growth rate on the embryo at competing for nutrients. This aspect deserves further studies.

### Effect of maternal milk yield level during embryogenesis on the offspring

In dairy cows, the fertilization of the egg and the first stages of embryonic development may occur in the vicinity of peak milk yield, which usually takes place between day 70 and 100 after calving. Thus, conception takes place in a metabolic environment characterized by either a mild negative, neutral, or slightly positive energy balance. The mode of the distribution of DIM at conception in the dam data set was 73, with 80% of conceptions occurring between 54 and 160 DIM. As there is a large variability (both phenotypic and genetic) for yield at the peak of lactation between cows (Table 2), we estimated the effect of milk production level of the lactating mother on the future performance (MY, DIM and FP) of the fetus.

**Table 2:** Classification of the mothers of the individuals according to milk production at conception.

Percentile group of mother's milk yield	Number of observations	Min daily milk (kg)	Max daily milk (kg)
**0 (non-lactating mother)** 1	16,288	-	-
**1 ( = <10^th^)**	2,384	5	22
**2 (>10^th^ & = <20^th^)**	3,369	22	25.8
**3 (>20^th^ & = <30^th^)**	2,435	25.8	28
**4 (>30^th^ & = <40^th^)**	2,808	28	30
**5 (>40^th^ & = <50^th^)**	3,097	30	32
**6 (>50^th^ & = <60^th^)**	2,999	32	34
**7 (>60^th^ & = <70^th^)**	2,579	34	36
**8 (>70^th^ & = <80^th^)**	3,337	36	38.5
**9 (>80^th^ & = <90^th^)**	2,864	38.5	42.4
**10 (>90^th^ & = <100^th^)**	2,903	42.4	60

1 individuals born from pregnancies in non lactating mothers (heifers) were grouped together (0), and all others were classified according to the tenth-percentiles of the milk production (kg) of the dam at conception.

**Figure 2:**
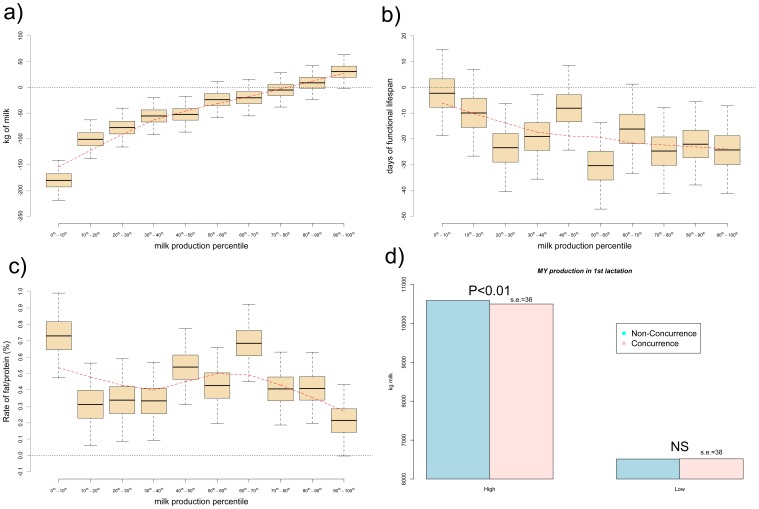
Effect of maternal production level during embryogenesis on adult performance. The boxplots show the effect of the maternal milk yield level (by percentiles) during embryogenesis in the performance of the adult individual for a) milk yield, b) lifespan and c) fat/protein. Values are expressed in relation to individuals whose embryogenesis occurred in the absence of maternal lactation. Boxes contain the lower and upper 25^th^ –percentiles of the posterior distribution and whiskers contain the respective highest probability density interval at 99%. The red-dashed line is the non-parametric regression of the performance of the individual on maternal milk yield level from the loess function in R [33]. d) The concurrence of maternal lactation during embryogenesis reduced the milk yield performance by 90 kg (P-value<0.01) in the adult life of females above the 90th percentile of additive genetic value, whereas it did not have effect on individuals with low genetic merit (in the lower 10th percentile).

Individuals whose first stages of fetal development occurred in the absence of maternal lactation produced 52 kg (MCs.e. = 0.009) more milk in their first lactation and lived 16 d (MCs.e. = 0.002) longer than the average cow born to mothers that conceived while lactating. However, contrary to our initial expectations, milk yield of these females was not significantly different from the most productive cows (daughters born to mothers with milk yield level above the 50^th^ percentile) in their first lactation. Not even daughters of mothers above the 90^th^ percentile for milk yield level produced significantly more milk than daughters of non-lactating mothers at conception (Figure 2.a). In addition, besides having similar production level than the daughters of most productive cows, the individuals that were gestated in the absence of her mother's lactation showed statistically significant longer lifespan than the daughters of multiparous females with high milk yield levels (Figure 2.b), although this antagonism between milk production and functional longevity in Holstein cows has been well documented previously [21]–[23].

On the other hand, individuals that were gestated during her mother's lactation and whose mother's milk yield were below the 50^th^-percentile of daily milk production showed lower milk yield in their first lactation but similar length of functional lifespan than individuals whose fetal development occurred in the absence of their mother's lactation (Figure 2.a and 2.b). Less productive cows usually present less metabolic diseases and better reproductive performance [24]–[26], and thus are at lower risk of culling and stay longer in the herds (longer functional lifespan).

Milk FP ratio in the first lactation was larger for individuals gestated during their mother's lactation, with a posterior distribution being larger than zero with probability 1 regardless the milk yield level of the mother at the time of conception.

The few studies that have previously evaluated the impact of milk performance of the dam on milking performance of the offspring have reported either a weak or no association between these two variables. The results herein are in contrast with those reported by Banos et al. [14], who observed no significant effects of maternal milk production during pregnancy on subsequent offspring milking performance in the first lactation. However, in line with results herein, Berry et al. [15] found a negative relationship between milk production of the dam and milking performance of the offspring in the first and third, but not second, lactations and concluded that the majority of the maternal effects in progeny performance were due to factors other than maternal milk production. The relative low number of animals (<20,000) included in the study from Banos et al. [14] could explain, partly, the inconsistent results. It has been also speculated that nutrition of the fetus during gestation might influence its fitness as an adult [27]. This is in agreement with the results of our study.

The negative effects of the concurrence of gestation and lactation on fetal programming was more noticeable in high-producing cows, as the energy mobilization and metabolic demands are greater in cows producing 46 kg of milk/day (classified as “High-producing”) than in cows yielding 20 kg of milk/day (classified as “Low-producing”). We selected 2 subsets of 4,507 individuals each: cows in the upper and lower 10^th^ percentiles of the estimated additive genetic values (**u**) distribution. We ran the same generalized linear model in each of the subsets adjusting by the same systematic effects (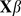) as described above, and included whether the fetal development of the individual coincided with the lactation of her mother (0 = non-concurrence/1 = concurrence). Females in the superior 10^th^ percentile for genetic merit were affected by the concurrence of the lactation of their mother during their fetal development, producing 90 (standard error, s.e. = 36) kg less milk than those females whose fetal development occurred in the absence of the maternal lactation. This effect was low, but statistically significant, with a *P*- value<0.01, whereas it was not the case in the group of cows in the lower 10^th^ percentile of the estimated additive genetic values distribution (estimate  = 4; s.e. = 38; NS) (Figure 2.d).

### Effects of mastitis during embryogenesis on the offspring

**Figure 3:**
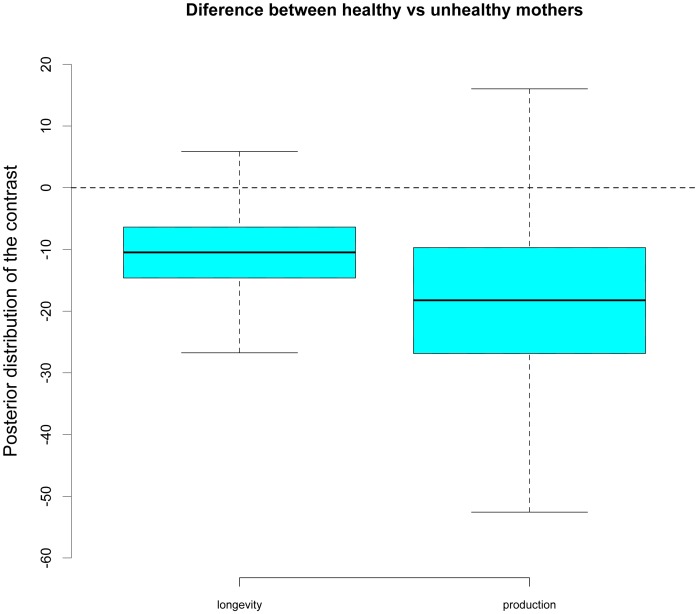
Consequences of the presence of maternal mastitis during embryo development. Boxes contain the lower and upper 25^th^ –percentiles of the posterior distribution of the contrast between females whose first stage of fetal development coincided with a maternal mastitis episode vs those from healthy mothers. Whiskers contain the highest probability density interval at 99% of such posterior distributions.

Mastitis is the most common diseases in dairy cattle [18], [20], [23]. It is characterized by bacterial infection and inflammation of the mammary gland, with a large increase in the presence of somatic cells in the milk (typically >400.000 cell/ml is considered as a case of clinical mastitis). We analyzed the effect of concurrence of maternal subclinical mastitis during embryonic and fetal development on adult performance. The number of females whose mothers presented subclinical mastitis during pregnancy was 5,846. Embryos developed during maternal mastitis episodes presented shorter productive lifespan than average (−11 days; MCs.e. = 8; HPD95 ranging between −22 and 2), with a slight (−18 kg; MCs.e. = 17), but non significantly different from zero, reduction of milk yield levels (HPD95 ranging from −43 to 7) as shown in Figure 3. A negative influence of maternal mastitis during embryogenesis was detected, although non-statistically significant evidence was found, probably due to the relatively low number of dams undergoing the disease (13%).

### Implications

It has been widely stated that maternal nutrition may play a role in the development of the fetus in mammalians (e.g. humans and mice). This study demonstrates that, at least in cattle, there are other factors, such as the presence of maternal lactation, the productive level of the mother or the concurrence of diseases (e.g. mastitis) during embryonic and fetal development that exert long-term effects on the offspring. Females gestated in non-lactating cows behave differently than females gestated in lactating cows, regardless of their genetic merit. So far, this effect has not been included in genetic evaluation models, and its inclusion poses some challenges such as the interaction between presence of maternal lactation and milk yield level. Management practices should carefully consider these aspects during the fetal development to raise healthier and more productive future individuals. Animal breeding has, so far, considered common environment to individuals as a whole, but the role of epigenetics on the GxE interaction should be considered [28]. Nowadays, genome-wide methylation status can be obtained, and a large amount of environmental information (environment-omics) is being recorded in farm animals, which may bring further insight on the genetic architecture of complex traits. Future studies should include observations on the genome and methylome of individuals to obtain evidences of potential epigenetic effects of maternal lactation during pregnancy in the progeny.

## Materials and Methods

### Data preprocessing

Data used were provided by the Spanish Holstein association and were extracted from the National milk recording scheme, which has been recording data since the later 1980's. Data consisted of first lactating cows rearing in three northern regions in Spain. Similar management and feeding practices are reported in the different regions, with some expected heterogeneity that was adjusted as described in the supplementary material (File S1). Cows that had any registered abortion before the first lactation or that stopped milking prior to reaching 1000 kg of milk were excluded from the analyses to avoid external noise in the models. Only cows with known pedigree and lactation records were used in the analyses. The lactation record for an individual provided information about date of birth, date of calving, lactation number, type of calving, milking records (daily kg of milk, fat, and protein, as well as fat and protein contents in milk), days in milk, date of end of lactation, calving interval, and complete identification for each cow and her parents. Only cows whose dam had available lactation records were considered. The final data set included 40,065 records from first lactating cows. Pedigree information was tracked as many generations back as possible, ending up with 131,308 individuals in the pedigree file. Selection of sires of cows in these regions are in accordance with worldwide breeding strategies, and typically worldwide top ranked sires are used as sire of cows [29].

The first ancestor generation of all animals with observations was 100% complete. The second ancestor generation was 99.8% complete. The average equivalent generation for the population of study was 10.67. The maximum number of known generations was 14. In the set of cows included in the analysis, the inbreeding coefficient (F) ranged from 0 to 37%, and its frequency distribution had a longer tail to the right. Mean, median and mode F were 3.9% 4% and 4%, respectively, with the 75th percentile of the distribution at 5%.

### Phenotypic values

Phenotypic measurements for milk, fat, and protein yields during first lactation were available of all 40,065 cows included in the analyses. The standardized production was considered, which is the corresponding yield for a lactation length of 305 days. This is a common adjustment of milk production in Holstein cattle used for fair comparison between animals with different lactation lengths. In addition, all individuals in the dataset had complete lactation records during their entire productive lifetime and the total amount of days in milk (productive days) in their lifetime was calculated.

Here, the phenotypic measurement of standardized milk production (MY) in the first lactation was chosen as the trait indicating production level of the animals, the ratio between standardized milk fat and protein yields (FP) was chosen as an indicator of metabolic function, and the total amount of days in milk (DIM) was chosen as a proxy for longevity.

### Models

Let 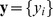 be the vector of the corresponding phenotypic values of the individuals for each of the indicator traits described above.

The following underlying statistical model was considered to obtain residual estimates from phenotypic observations after adjusting by environmental circumstances and genetic effects [17], [30], [31]. A detailed description of the adjustment is provided in the supplementary material (File S1).
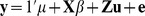



Then, the posterior mean estimates of the residuals 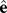 are expected to be estimates of the performance of the individuals that are independent any environmental circumstance (such as feeding strategies, management practices or physiological status of the individual generating the data), and genetic selection and genetic merit of individuals. These residual estimates were used as a dependent variable to estimate the effect of different maternal circumstances (**m**) during conception in the future adult individual as:
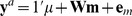
Where 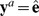 are the posterior mean estimates of the residuals of the adjustment of the phenotypic records by environmental and genetic effects, *μ* is a sample mean, **1** is a column vector of ones, and **e** *_m_* are the residuals after adjusting by **m**. The maternal circumstances were considered in separate models and were respectively: concurrence of maternal lactation (4 levels: 0-non concurrence, 1-concurrence with the first maternal lactation, 2-concurrence with the second maternal lactation, 3-concurrence with the third or subsequent maternal lactation), milk yield level of the mother at fecundation (11 levels: 0-no milk production, 1–10 corresponding to the 10^th^ percentile of milk yield production in the population) at fecundation, or occurrence of fecundation during a mastitis disease episode (2 levels: 0-no disease, 1-disease).

A hierarchical Bayesian model was implemented to estimate the effect of the environmental circumstances.

Likelihood: 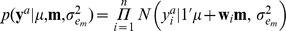


Prior: 
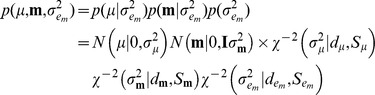


Conditional distributions of these effects were obtained using Monte-Carlo Markov chains, as:
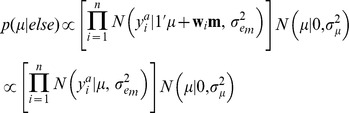
This is recognized as the kernel of a normal distribution with mean 
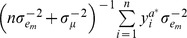
 and variance 
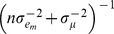
, where 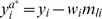.
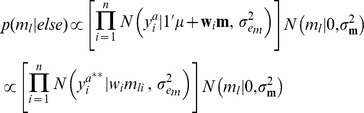



This is recognized as the kernel of a normal distribution with mean and variance equal to the solution of 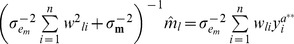, where 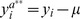.

In practice, 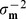 can be set large enough so that an efficient effectively flat prior is assigned to these coefficients.
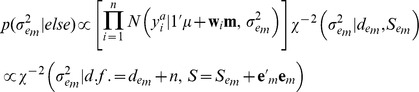
where 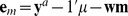.

The analyses were based on a single chain of 10,000 iterations, with the first 1,000 samples discarded. The posterior distributions for the contrast of m regarding level 0 (absence of maternal lactation during first stages of fetal development) were obtained. Monte-Carlo standard errors were calculated for the mean of the posterior distributions of the contrasts [32], and HPD95 were considered to range between the corresponding 2.5^th^ and 97.5^th^ quantiles.

## Supporting Information

File S1 **Supplemental statistical procedure information.**
(DOCX)
