# Supplementary material for: Trans-Generational Effect of Maternal Lactation during Pregnancy: A Holstein Cow Model
Source: PLoS One. 2012 Dec 20;7(12):e51816. doi: 10.1371/journal.pone.0051816 (PMC3527476; doi:10.1371/journal.pone.0051816)
Supplement: File S1 — Supplemental statistical procedure information. (DOCX) [file pone.0051816.s001.docx]

# File S1. *Supplemental statistical procedure information*.

The following underlying statistical model was considered:

$$\mathbf{y}=\mathbf{1}'\mu+\mathbf{X}\beta+\mathbf{Zu}+\mathbf{e}$$

here, the *i*th component of the *n*-vector **y** depicts the phenotypic value of individual *i*. The **β** vector corresponds to the environmental forces affecting the trait, with **X** being the corresponding incidence matrix. Then, $\mathbf{u}=\left\{u_{i}\right\}$ represents a vector of additive genetic effects, with $u_{i}$ being the additive genetic merit of animal *i* in the pedigree. A priori, the additive genetic effects were assumed to be distributed as , with $\mathbf{G}=\mathbf{A}\sigma_{a}^{2}$, where **A** is a (*txt*) additive relationship matrix between animals, with *t*=131,308, and $\sigma_{a}^{2}$ corresponds to the additive genetic variance. The matrix **Z** is an (*nxg*) incidence matrix, which rows consist of unit vectors with one component being 1 and all the others zero, indicating the respective position of the individual with record in the *g*-vector of genetic merit of all individuals in the relationship matrix.

The residuals **e** were assumed to be distributed as $N(\mathbf{0},\sigma_{e}^{2})$, where $\sigma_{e}^{2}$ is the residual variance

A hierarchical Bayesian model was implemented to estimate the residuals of the model as described next:

Likelihood: $p\left(\mathbf{y}|\mu,\beta,\mathbf{u},\mathbf{G},\sigma_{e}^{2}\right)=\prod_{i=1}^{n} N\left(y_{i}|\mu+\mathbf{x}_{i}\beta+\mathbf{z}_{i}\mathbf{u},\sigma_{e}^{2}\right)$

Prior:

$$\begin{matrix} p\left(\mu,\beta,\mathbf{u},\mathbf{G},\sigma_{e}^{2}\right)=p\left(\mu|\sigma_{e}^{2}\right)p\left(\beta|\sigma_{e}^{2}\right)p\left(\mathbf{u}|\mathbf{G}\right)p\left(\mathbf{G}\right)p\left(\sigma_{e}^{2}\right) \\ =N\left(\mu|0,\sigma_{\mu}^{2}\right)N\left(\beta|0,\mathbf{I}\sigma_{\beta}^{2}\right)N\left(\mathbf{u}|0,\mathbf{G}\right) \\ \times\chi^{-2}\left(\sigma_{\mu}^{2}|d_{\mu},S_{\mu}\right)\chi^{-2}\left(\sigma_{\beta}^{2}|d_{\beta},S_{\beta}\right)\chi^{-2}\left(\mathbf{G}|d_{u},S_{u}\right)\chi^{-2}\left(\sigma_{e}^{2}|d_{e},S_{e}\right) \end{matrix}$$

Above, $N\left(y_{i}|\mu+\mathbf{x}_{i}\beta+\mathbf{z}_{i}\mathbf{u},\sigma_{e}^{2}\right)$, $N\left(\mu|0,\sigma_{\mu}^{2}\right)$, $N\left(\beta|0,\mathbf{I}\sigma_{\beta}^{2}\right)$, and $N\left(\mathbf{u}|0,\mathbf{G}\right)$ are normal densities centered at $\mu+\mathbf{x}_{i}\beta+\mathbf{z}_{i}\mathbf{u}$, or 0, with variances $\sigma_{e}^{2}$, $\sigma_{\mu}^{2}$, $\sigma_{\beta}^{2}$ and **G**, respectively. $\chi^{-2}\left(\sigma_{e}^{2}|d_{e},S_{e}\right)$, $\chi^{-2}\left(\sigma_{\mu}^{2}|d_{\mu},S_{\mu}\right)$, $\chi^{-2}\left(\sigma_{\beta}^{2}|d_{\beta},S_{\beta}\right)$ and $\chi^{-2}\left(\mathbf{G}|d_{u},S_{u}\right)$ are scaled-inverted chi-square densities, with $d_{\cdot}$ degrees of freedom and scale $S_{\cdot}$, respectively. The role of **u** is to adjust the additive action of the genes inherited in the individual by means of a co-variance matrix between individuals using the rules of the numerator relationship matrix [1]. In this model, all fully conditionals have closed form, thus a Gibbs sampler algorithm can be used to draw samples from the joint posterior distributions [2], with standard derivations are then used for Bayesian linear models [3]. The joint posterior distribution is:

$$\begin{matrix} p\left(\mu,\beta,\mathbf{u},\mathbf{G},\sigma_{e}^{2}|\mathbf{y}\right)=\prod_{i=1}^{n} N\left(y_{i}|\mu+\mathbf{x}_{i}\beta+\mathbf{z}_{i}\mathbf{u},\sigma_{e}^{2}\right) \\ \times N\left(\mu|0,\sigma_{\mu}^{2}\right)N\left(\beta|0,\mathbf{I}\sigma_{\beta}^{2}\right)N\left(\mathbf{u}|0,\mathbf{G}\right) \\ \times\chi^{-2}\left(\sigma_{\mu}^{2}|d_{\mu},S_{\mu}\right)\chi^{-2}\left(\sigma_{\beta}^{2}|d_{\beta},S_{\beta}\right)\chi^{-2}\left(\mathbf{G}|d_{u},S_{u}\right)\chi^{-2}\left(\sigma_{e}^{2}|d_{e},S_{e}\right) \end{matrix}$$

The fully conditional distribution of any unknown is obtained by removing from the right-hand side of the equation above the components that do not involve such an unknown [3]. The remaining components are kernels of known distribution as conjugate priors were chosen.

The fully conditional distribution for each unknown is given next.

1. Intercept

$$\begin{matrix} p\left(\mu|else\right)\propto\left[\prod_{i=1}^{n} N\left(y_{i}|\mu+\mathbf{x}_{i}\beta+\mathbf{z}_{i}\mathbf{u},\sigma_{e}^{2}\right)\right]N\left(\mu|0,\sigma_{\mu}^{2}\right) \\ \propto\left[\prod_{i=1}^{n} N\left(y_{i}^{*}|\mu,\sigma_{e}^{2}\right)\right]N\left(\mu|0,\sigma_{\mu}^{2}\right) \end{matrix}$$

This is recognized as the kernel of a normal distribution with mean $\left(n\sigma_{e}^{-2}+\sigma_{\mu}^{-2}\right)^{-1}\sum_{i=1}^{n} y^{*}_{i}\sigma_{e}^{-2}$ and variance $\left(n\sigma_{e}^{-2}+\sigma_{\mu}^{-2}\right)^{-1}$, where $y_{i}^{*}=y_{i}-\mathbf{x}_{i}\beta-u_{i}$.

2. Regression coefficients for environmental effects ()

$$\begin{matrix} p\left(\beta_{j}|else\right)\propto\left[\prod_{i=1}^{n} N\left(y_{i}|\mu+\mathbf{x}_{i}\beta+\mathbf{z}_{i}\mathbf{u},\sigma_{e}^{2}\right)\right]\times N\left(\beta_{j}|0,\sigma_{\beta}^{2}\right) \\ \propto\left[\prod_{i=1}^{n} N\left(y_{i}^{**}|\mathbf{x}_{i}\beta_{j},\sigma_{e}^{2}\right)\right]N\left(\beta_{j}|0,\sigma_{\beta}^{2}\right) \end{matrix}$$

This is recognized as the kernel of a normal distribution with mean and variance equal to the solution of $\left(\sigma_{e}^{-2}\sum_{i=1}^{n} x_{ij}^{2}+\sigma_{\beta}^{-2}\right)^{-1}\hat{\beta}_{j}=\sigma_{e}^{-2}\sum_{i=1}^{n} x_{ij}y_{i}^{**}$, where $y_{i}^{**}=y_{i}-\mu-\sum_{p\neq j} x_{ip}\beta_{p}-u_{i}$. In practice one can set $\sigma_{\beta}^{2}$ large enough so that an efficient effectively flat prior is assigned to these coefficients.

3. Infinitesimal additive genetic effect (**u**)

$$p\left(\mathbf{G}|else\right)\propto N\left(\mathbf{u}|0,\mathbf{G}\right)\times\chi^{-2}\left(\mathbf{G}|d_{u},S_{u}\right)$$

This is recognized as a multivariate normal distribution with mean vector (co-variance matrix) equal to the solution of the system, $\left(\mathbf{I}\sigma_{e}^{-2}+\mathbf{G}^{-1}\right)^{-1}\hat{\mathbf{u}}=\sigma_{e}^{-2}\mathbf{y}^{***}$, where $y_{i}^{***}=y_{i}-\mu-\mathbf{x}_{i}\beta$.

4. Infinitesimal additive genetic variance ($\sigma_{a}^{2}$)

Given that $\mathbf{G}=\mathbf{A}\sigma_{a}^{2}$

$$p\left(\mathbf{G}|else\right)\propto N\left(\mathbf{u}|0,\mathbf{G}\right)\times\chi^{-2}\left(\mathbf{G}|d_{u},S_{u}\right)$$

or equivalently,

$$\begin{matrix} p\left(\sigma_{a}^{2}|else\right)\propto N\left(\mathbf{u}|0,\sigma_{a}^{2}\right)\times\chi^{-2}\left(\sigma_{a}^{2}|d_{a},S_{a}\right) \\ \propto\chi^{-2}\left(\sigma_{a}^{2}|d.f.=d_{a}+t,S=S_{a}+\mathbf{u}'\mathbf{A}^{-1}\mathbf{u}\right) \end{matrix}$$

where *t* is the order of the square matrix **A**.

5. Residual variance ()

$$\begin{matrix} p\left(\sigma_{e}^{2}|else\right)\propto\left[\prod_{i=1}^{n} N\left(y_{i}|\mu+\mathbf{x}_{i}\beta+\mathbf{z}_{i}\mathbf{u},\sigma_{e}^{2}\right)\right]\times\chi^{-2}\left(\sigma_{e}^{2}|d_{e},S_{e}\right) \\ \propto\chi^{-2}\left(\sigma_{e}^{2}|d.f.=d_{e}+n,S=S_{e}+\mathbf{e}'\mathbf{e}\right) \end{matrix}$$

where $\mathbf{e}=\mathbf{y}-\mathbf{1}'\mu-\mathbf{x}_{i}\beta-\mathbf{z}_{i}\mathbf{u}$.

**References for S1**

1. Henderson CR (1976) A Simple Method for Computing the Inverse of a Numerator Relationship Matrix Used in Prediction of Breeding Values. Biometrics 32 (1): 69–83. [DOI](http://en.wikipedia.org/wiki/Digital_object_identifier):[10.2307/2529339](http://dx.doi.org/10.2307%2F2529339)
2. Gelfand A, Smith AFM (1990) Sampling based approaches to calculating marginal densities. J. Anim. Stat. Assoc. 85: 398-409.
3. Sorensen DA, Gianola D (2002) *Likelihood, Bayesian and MCMC Methods in Quantitative Genetics*. Springer-Verlag, New York, pp. 588-595.
